# Contribution of the co.LAB Framework to the Collaborative Design of Serious Games: Mixed Methods Validation Study

**DOI:** 10.2196/33144

**Published:** 2021-11-24

**Authors:** Dominique Jaccard, Laurent Suppan, Félicia Bielser

**Affiliations:** 1 Media Engineering Institute HES-SO University of Applied Sciences and Arts Western Switzerland Yverdon Switzerland; 2 Division of Emergency Medicine Department of Anesthesiology, Clinical Pharmacology, Intensive Care and Emergency Medicine University of Geneva Hospitals and Faculty of Medicine Geneva Switzerland; 3 School of Health Sciences (HESAV) HES-SO University of Applied Sciences and Arts Western Switzerland Lausanne Switzerland

**Keywords:** serious game, educational game, education, simulation game, gaming, design, framework, methodology, mixed method, validation

## Abstract

**Background:**

Multidisciplinary collaboration is essential to the successful development of serious games, albeit difficult to achieve. In a previous study, the co.LAB serious game design framework was created to support collaboration within serious game multidisciplinary design teams. Its use has not yet been validated in a real usage context.

**Objective:**

The objective of this study was to perform a first assessment of the impact of the co.LAB framework on collaboration within multidisciplinary teams during serious game design and development.

**Methods:**

A mixed methods study was conducted, based on 2 serious game design projects in which the co.LAB framework was used. The first phase was qualitative and carried out using a general inductive approach. To this end, all members of the first serious game project team who used the co.LAB framework were invited to take part in a focus group session (n=6). In a second phase, results inferred from qualitative data were used to define a quantitative instrument (questionnaire) that was designed according to the Checklist for Reporting Results of Internet E-Surveys. Members of both project teams (n=11) were then asked to answer the questionnaire. Quantitative results were reported as median (Q1, Q3), and appropriate nonparametric tests were used to assess between-group differences. Finally, results gathered through the qualitative and quantitative phases were integrated.

**Results:**

In both phases, the participation rate was 100% (6/6 and 11/11). Verbatim transcripts were classified into 4 high level themes: (1) influence on collaborative dimensions; (2) impact on project course, monitoring, and efficiency; (3) qualitative perceptions of the framework; and (4) influence of team composition on the use of the framework. The web-based questionnaire was then developed according to the 7 dimensions of collaboration by Burkhardt et al. In both projects, the co.LAB framework had a positive impact on most dimensions of collaboration during the multidisciplinary design and development of serious games. When all collaborative dimensions were aggregated, the overall impact of the framework was rated on a scale from –42 to 42 (very negative to very positive). The overall median score was 23 (Q1, Q3: 20, 27), with no significant difference between groups (*P*=.58). Most respondents also believed that all serious game design teams should include a member possessing significant expertise in serious game design to guide the development process.

**Conclusions:**

The co.LAB framework had a positive impact on collaboration within serious game design and development teams. However, expert guidance seems necessary to maximize development efficiency. Whether such guidance can be provided by means of a collaborative web platform remains to be determined.

## Introduction

### Background

Efficient multidisciplinary collaboration is essential to the successful development of serious games [1-4]. This collaboration can, however, prove difficult to achieve, as members of the development team come from different backgrounds and might have divergent expectations [4-7]. Communication and collaboration could be enhanced by using comprehensive design frameworks, the aim of which is to guide development teams during serious game design and development [4,7,8].

### The co.LAB Methodological Framework

Funded by the Swiss National Science Foundation, the aim of the project “co.LAB - A Digital Lab for the co-Design, co-Development and co-Evaluation of Digital Learning Games” (co.LAB) is to improve efficiency and relevance in serious game design and development by supporting the collaboration between multidisciplinary development teams.

This project has already led to the creation and publication of the co.LAB generic serious game design framework (co.LAB framework) [9]. In their article, authors of the co.LAB framework stated that it was defined based on a literature review and on its authors' experiences. But the co.LAB framework has not been validated in a naturalistic context yet.

**Figure 1:**
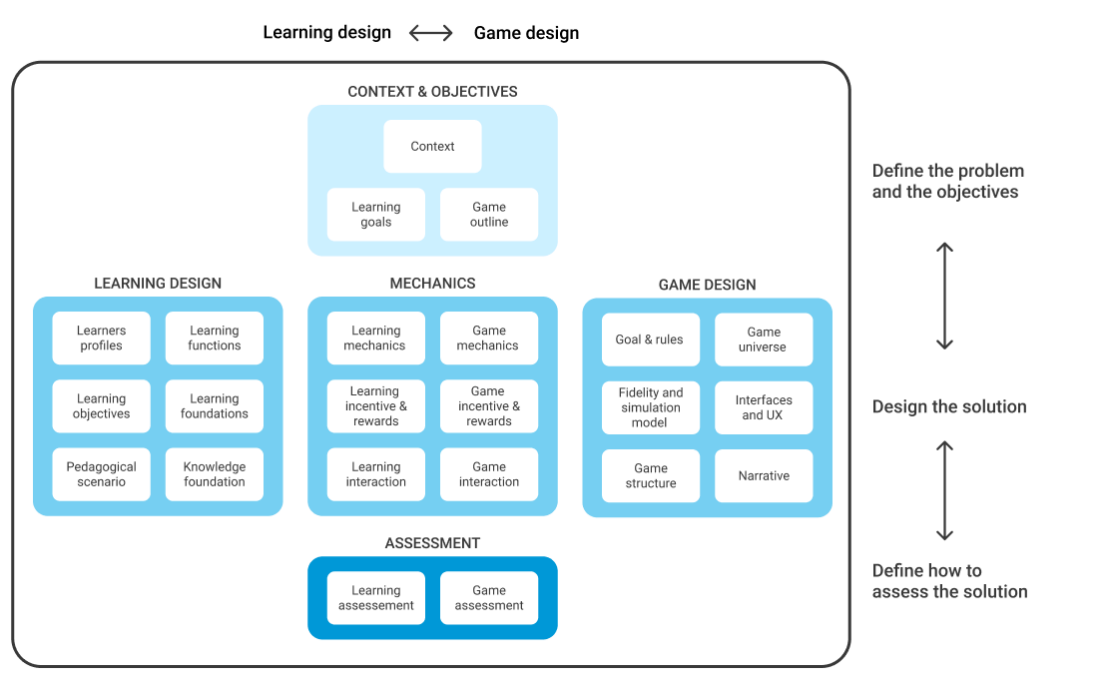
The co.LAB generic framework for serious game design (adapted from [9]). UX: user experience.

While most existing frameworks are dedicated to the design and development of specific types of educational games [4,10], the co.LAB framework was conceived as an adaptive framework that should allow the design of a wide range of educational games [9]. The co.LAB framework contains 5 main categories (“context and objectives,” “game design,” “mechanics,” “learning design,” and “assessment”) and was designed to provide multidisciplinary teams with a global understanding of the design process. Different views of the framework can be used to help team members apprehend the different components of serious games design (Figure 1).

The horizontal view (either left-to-right or right-to-left) of the co.LAB framework emphasizes that serious game design is a blend of learning and game design, with the Mechanics category representing the link between the game and learning. In the vertical view, the co.LAB framework can be seen under a project management perspective: The upper section defines project objectives, the middle section defines the solution (the game and the associated learning concept), and the bottom section defines how the solution will be evaluated both from the game and learning perspectives [9].

To date, and to the best of our knowledge, the co.LAB framework has been used for serious game design and development by 2 multidisciplinary development teams.

### Objectives

The objective of this study was to perform a first assessment of the impact of the co.LAB framework on collaboration within multidisciplinary teams during serious game design and development. The following research question was formulated: How does a multidisciplinary team perceive the contribution of the co.LAB framework to the collaborative design and development of serious games?

## Methods

### Overview

In order to better understand the contribution of the co.LAB framework to collaboration, mixed methods research with an exploratory design was conducted. The following phases were carried out sequentially [11]: (1) qualitative phase (focus group [FG]): qualitative data collection, data analysis, results; (2) mixing phase: development of an instrument to allow quantitative measurements; (3) quantitative phase (questionnaire): quantitative data collection, data analysis, results; (4) interpretation phase: integration of quantitative and qualitative results, generalization.

#### Projects and Participants

The study included team members of the first 2 projects in which the co.LAB framework was used.

The first project, Patients’ Rights and Innovative Teaching Strategies (PRITS) [12], aimed at designing and developing a serious game to support health students in learning about patient rights. The project team included 2 professors of law (lawyers specializing in patients’ rights), 1 health care lecturer, 1 educational researcher, 1 serious game expert, 1 graphic designer, and 2 computer scientists. The project was led by the School of Health Sciences (HESAV) and Media Engineering Institute (MEI), both at the University of Applied Sciences of Western Switzerland (HES-SO). The project began in September 2020 and ended in August 2021. It was thus almost completed at the time of this study (June 2021).

The second project, Interprofessional Major Incident Simulator (InterMIS), was a large-scale project aiming at developing a serious game to train health professionals to manage exceptional events. The project team includes 3 medical doctors, 2 paramedic instructors, 1 serious game expert, 1 graphic designer, and 2 computer scientists. This project is led by MEI at HES-SO and the Geneva University Hospitals. The project began in January 2021 and should span over 4 years. At the time of this study (June 2021), the project was at the end of the design phase, which included the design of both the learning and game aspects.

#### Experimental Conditions of the Uses of the co.LAB Framework

In both projects (PRITS and InterMIS), the co.LAB framework was used since inception. The framework was reproduced into an online shared folder. All team members had access to the overview of the framework and to their specific project folder. The use of the co.LAB framework for the design and development of both projects was chosen by the development leader. Therefore, participants had to assess the impact of a framework that they had not chosen and had not used before. Thus, an early adopter effect can hardly be held accountable for the results obtained in the course of this study.

### Focus Group

In this first qualitative phase, a general inductive approach as described by Thomas [13] was chosen. This approach was considered appropriate in this context as it allows for the simple inference of findings from data derived from focused questions and discussions [13]. Moreover, FGs are particularly suitable for exploring people’s knowledge and daily experiences [14,15] and for exploring ideas and opinions operating through communication [16].

#### Procedures

The FG was conducted according to the Consolidated Criteria for Reporting Qualitative Research (COREQ) guidelines [17] (Multimedia Appendix 1). An FG interview guide was established (Multimedia Appendix 2). The questions stated in this FG interview guide are only intended to give an overall idea of the questions actually asked during the FG. Indeed, as acknowledged by Krueger [18], questions are often asked in a somewhat different way than reported in such general guides.

An FG, which lasted 1.5 hours, was held at HESAV and recorded using an audio device. Participants (n=6) were asked to discuss their use of the co.LAB framework during their serious game design and development activities (individual and collective) and to explore its impact on collaboration.

Participants provided their consent to the recording and use of FG data for research purposes. The FG was held by an educational scientist with previous experience in conducting qualitative research and moderating such groups. One of the authors of the co.LAB framework introduced the FG and attended the FG as an external observer.

#### Participants

As one of the team members was the main author of the co.LAB framework, he did not act as a participant during the FG session but was nevertheless present as an observer. A second team member participated in the definition of the assessment of the contribution of the co.LAB framework to collaboration and acted as a facilitator rather than as a participant. Of the 6 members of the PRITS project who attended the FG, 3 were from the learning design and development side of the co.LAB framework (1 project owner and 2 professors of law), and 3 were from the game design and development side of the co.LAB framework (2 computer scientists and 1 graphic designer).

#### Data Analysis

A thematic analysis was performed on the data to identify themes. The thematic analysis was based on the process described by Braun and Clarke [19], with the steps outlined in Textbox 1.

**Steps followed during the thematic analysis to identify themes.**

1. Transcription:
  A “clean read” transcript was created based on the audio recording.
  Based on the transcript, a summary of the discussion between participants was generated in connection with the questions.
2. Coding
  Verbatim transcripts were coded with the qualitative analysis software MAXQDA (VERBI Software GmbH, Berlin, Germany).
3. Searching for themes
  Themes were generated from codes.
4. Reviewing and defining themes
  Themes were grouped into high-level themes.
5. Producing the report
  A write-up of each theme content was performed, and representative quotes were selected.
  As the focus group took place in French, we did an automatic English translation of selected quotes with DeepL Translate (DeepL GmbH, Köln, Germany). We chose to do an automatic translation as it guarantees the reproducibility and reduces the risks of interpretation by authors while translating. This automatic translation was proofread by an author and validated by a second author.

In addition, the team dynamic during the FG was analyzed, and agreements and disagreements between participants were identified and coded.

Each phase was initially carried out by one author, then confirmed by another. Along the process, authors in charge of the initial proposition were alternated. Any disagreement was resolved by reaching consensus.

### Web-Based Questionnaire

Based on the FG results, a 3-tiered, web-based questionnaire was developed following Eysenbach’s Checklist for Reporting Results of Internet E-Surveys (CHERRIES) [20] (Multimedia Appendix 3).

#### Questionnaire Design

The first part was designed to gather sociodemographic data, including prior serious game design experiences and roles held in these projects.

The second part focused on the contribution of the co.LAB framework to the collaboration. This part was constructed based on the 7 dimensions of collaboration proposed by Burkhardt et al [21]. The authors’ original version was intended to assess the quality of the collaboration within a team. Since our goal was to evaluate the contribution of a methodology as a support to collaboration, we developed questions designed to specifically assess the contribution of the co.LAB framework. For each of the dimensions, we developed 3 questions, with one of them being formulated as a reversed item. The questions were proposed by a first author, completed by a second author, and validated by all authors.

In the third part, we added questions related to elements of collaboration and usability of the co.LAB framework that emerged from the FG analysis.

A translated version of this questionnaire is available (Multimedia Appendix 4)**.**

#### Web-Based Platform

The questionnaire was hosted on a web platform created under the Joomla! 3.9 content management system (Open Source Matters, New York, NY) [22]. It was created using the Community Surveys 5.6 component (Corejoomla, Hyberabad, India) and administered to a convenience sample of 11 participants: 6 from the PRITS project and 5 from the InterMIS project. The web platform was protected by 2 different software firewalls: RSFirewall 3 (RSJoomla, Constanta, Romania) and Admin Tools 6 (Akeeba Ltd, Nicosia, Cyprus). To avoid potential double entries, participants were required to fill in a short registration form before they could access the survey. All data were automatically recorded and securely stored in an encrypted MySQL-compatible database (MariaDB 5.5.5; MariaDB Foundation, Wakefield, MA) hosted on a Swiss server.

Answers to multiple answer and multiple-choice questions were mandatory, and all questions had to be completed before participants were allowed to proceed to the next page. Answers could be modified until the questionnaire was marked as completed (after clicking the “Finish” button). Questionnaires could be resumed if participants were disconnected or chose to log out temporarily.

The platform, registration form, questionnaire, and data extraction mechanism were thoroughly tested by all co-authors prior to the quantitative phase. Only then were participants invited, by email, to complete the questionnaire. No incentive was provided to encourage participants to complete the questionnaire.

#### Data Extraction and Statistical Analysis

Data were extracted from the MariaDB database to a CSV file. It was then imported and curated under Stata 16.1 (Statacorp LLC, College Station, TX). Given the limited sample size (n=11), data are presented as median (Q1, Q3) rather than as mean (SD). Questionnaires containing inconsistent answers were excluded. Inconsistent answers were detected when either the maximum (5) or minimum value (1) was given to all 3 questions assessing a specific dimension of collaboration as each dimension contained inversely phrased questions.

Answers based on 5-point Likert scales were then ascribed numerical values. Neutral answers (eg, “neither agree nor disagree”) were given a value of 0, answers backing the use of the co.LAB framework were given positive values (either 1 or 2), and answers opposing it were given negative values (either –1 or –2).

Each question regarding the 7 dimensions of collaboration was first analyzed separately. Then, the 3 questions belonging to each specific dimension were grouped, thereby generating a score ranging from –6 to 6. Finally, all 21 questions assessing the 7 dimensions by Burkhardt et al [21] were pooled to give an overall representation of the framework’s impact on a score ranging from –42 to 42. All questions were evenly weighted.

The answers to Likert-based questions assessing other elements of collaboration were assigned points ranging from 1 (for “Very small/none”) to 5 (for “Very high”).

Nonparametric tests were used to assess differences between the InterMIS and PRITS groups. Individual questions were assessed using Fisher exact tests, and the Mann-Whitney U test was used to evaluate pooled results (overall score and scores by dimension). A *P* value <.05 was considered significant.

### Consent and Institutional Review Board

All participants were provided with project information and gave their consent to participate and to the use of data for research purposes.

According to the Swiss Human Research Act [23], institutional review board approval was not necessary for this study, as participants did not belong to a vulnerable population and there was no medical intervention whatsoever.

## Results

### Focus Group

The classification of the verbatim transcripts enabled us to cluster the discussions about the contribution of the co.LAB framework into 4 high-level themes. These high-level themes, along with their subthemes, can be seen in Table 1.

**Table 1:** Themes and subthemes inferred from the focus group discussions.

High-level themes	Subthemes
Influence on collaborative dimensions	Fluidity of collaboration, information exchange for problem solving, argumentation and reaching consensus, sustaining mutual understanding, individual task orientation, task and time management
Impact on project course, monitoring, and efficiency	Use at all stages of the project, the project’s common thread, role clarification, monitoring, common objectives, structuring, outcomes related to the framework
Qualitative perceptions of the framework	Positive perceptions, negative perceptions
Influence of team composition on the use of the framework	Team size, team experiences, team quality and diversity, team autonomy in using the framework

The next sections provide a summary of each theme, with a few quotes selected for their representativeness. The final section presents the results of the team dynamics analysis.

#### Influence on Collaborative Dimensions

The co.LAB framework positively influenced 6 of the 7 dimensions of collaboration proposed by Burkhardt et al[21]. Participants found that the fluidity of collaboration was supported by this framework, as each member knew both what to do and what the others were to do. Consensus was more easily reached, as each member was able to react to what had been written or proposed by others (this was achieved by using shared files embedding collaborative features). Information sharing was promoted by granting access to centralized and structured information. Personal motivation was bolstered by making it possible to monitor the progress of the project. Mutual understanding was strengthened by a clear definition of the roles and using a common terminology. Finally, management of time and activities was facilitated by allowing an almost permanent and real-time overview of the project progress and of the tasks that still had to be performed.

Regarding fluidity of collaboration, one participant emphasized what was previously said by others, as follows:

The method helps with the fluidity of collaboration because you know who is doing what; it makes things clearer for everyone. The fluidity of collaboration is what impressed me the most for the reasons already mentioned.

Designer

The co.LAB framework was implemented in a shared online environment where team members could find the information. A participant who joined the development team while the project was already underway expressed how the framework had helped her assimilate information previously shared by and between team members:

I came in during the course of the project, so it allowed me to get into the flow of the previous information. When you come into a project, it's always extremely difficult to know what has been discussed. I appreciated having a place where everything was brought together in a clear way.

Developer 2

Another participant acknowledged that “we are forced to reach consensus” (Developer 1). This participant considered that the framework reproduced in shared files helped team members to find agreement because it allowed them to see what others had written and gave them the possibility to react accordingly.

A participant pointed out that a common understanding was promoted by the common terminology established by virtue of the framework:

For mutual understanding, this method allows us to have common terms, to know what we are talking about and where we stand.

Professor of law 1

Mutual understanding was also strengthened by clarifying the respective roles while setting common objectives:

It's allowed for common goals, without getting lost, to guide our work. Especially when there are a lot of us, coming from different fields.

Professor of law 1

The framework also allowed people from different disciplines to give their opinion and to take notice of those from others:

In a project with multiple disciplines, it helped to know when we had a voice. It's not forbidden to speak, but we knew when we had more legitimacy, on which points of co.LAB we could contribute more, and when we should stay in the background, leave the place to the person who has the competence, rather than having everyone throw in their two cents, not necessarily at the best time.

Developer 1

The framework was seen as having the potential to positively influence motivation:

For motivation, I think it helps indirectly. It wasn't the case, but if I had had a drop in motivation, I think that going back to the framework and seeing the progress, what's going on in green, would have helped me get motivated again.

Designer

The methodology also positively influenced fluid and spontaneous task allocation between project members:

As the main applicant, I was concerned about making sure we got to the end of the project, that all the tasks were done. But I didn't have to impose anything. People assigned themselves the tasks naturally, and my role was comfortable.

Project Owner

#### Impact on Project Course, Monitoring, and Efficiency

Using the co.LAB framework was perceived as having a positive impact on the course of the project. It was used from inception and all along the project. Throughout the project, it was perceived as a guide, a common thread. In that way, the co.LAB framework was considered as a supporting structure for the design and development process and for project monitoring. The use of the co.LAB framework was also perceived as having a positive impact on efficiency. Participants shared their thoughts on using this methodology in other contexts, for example without an expert or in another team configuration, but there were no responses showing a negative impact of the framework on collaborative work.

All participants answered that the framework was used as soon as they joined the project and then throughout it. A participant said that she had even already used it for a project proposal:

We used it from the very beginning and already in the preparation of the project proposal.

Professor of law 2

Several participants agreed about the use of co.LAB framework throughout the project and its role as a guideline and project monitoring tool:

If I remember correctly, each meeting included the milestones of the methodology. It gave us a roadmap to follow. It really made an impact because it structured our meetings, allowing us to know what we had to pay attention to for the next sessions. Beyond the tools, the organizational aspect of the methodology was very comfortable. It gave a vision of the next steps.

Project Owner

The co.LAB framework was seen as enabling people to better understand what to do, in a consistent way across disciplines:

On the development side, it helped to know what mechanics to put in place. I wasn't present at every meeting, and going to co.LAB was a way to know if we needed to implement a button or something else. It was a way to get direction, not to do something that was not discussed, not to do something that wasn’t planned: not to do too much, but to do the right thing, to refocus.

Developer 1

Team members frequently used the word “structure,” which illustrates their perception of the framework as assembling parts of a whole. The project was considered to have been carried out effectively by virtue of the structuring effect and of the centralization of information provided by the co.LAB framework:

It was a structure that made it possible to work quickly and well.

Developer 1

The structure is nice. With the centralized information, I could go and look for what had to do with IT development. Nothing bothered me; on the contrary, I could focus on the points that were important to me. Sometimes there were meetings where I felt less concerned, but in the end it's not such a bad thing; it helps to understand the project. Without the help of the co.LAB method, we might not have had this efficiency.

Developer 1

The project owner raised a question about the context of use of the framework in larger teams:

It's more of a question: We only had 4 disciplines and a small group (8 people). Can we transpose this methodology to a larger group with more disciplines?

Project Owner

#### Qualitative Perceptions of the Framework

Team members were asked to discuss positive and negative characteristics of the co.LAB framework. The discussion showed a clear predominance of positive aspects, with terms such as comfortable, clear, reassuring, liked, user-friendly, easy, understandable, and pleasant. These positive aspects are linked to most of the themes already presented: information sharing, project monitoring, structure, or guidelines. The only negative aspect was the fact that the content was mainly introduced and synthesized by one person, who guided the project.

Usability and simplicity were seen as factors favoring the adoption of the framework:

I liked it because it's a user-friendly tool. It's simple to use, logical to follow. This ease of use allows the method to be adhered to.

Project Owner

It was perceived as a convenient source of information, both for internal and external uses:

It's quite comfortable to bring out information among others for our funders. It has been an essential source of information for me.

Project Owner

One team member reported positive aspects of the framework, as follows:

The method is very clear. And there was availability, a nice collaboration, and I think the method helps in that.

Professor of law 1

On the negative side, a participant said that she was involved in creating the content but had limited participation at other levels:

On the negative aspects, I did not complete the content. We were involved in the content but not in proposing the steps.

Professor of law 2

#### Influence of Team Composition on the Use of the Framework

All team members described themselves as having no previous experience in serious game design nor had they heard about the co.LAB framework before starting the project. As such, they pointed out the value of having a team member (who did not participate in the FG) with expertise in serious game design and who had extensive knowledge of the co.LAB framework. The presence and role of this expert were seen as a success factor for the use of the framework. It raised a question about the importance of having an expert and whether the framework could be used without guidance.

Regarding the benefits of having an expert of the framework among the team, a professor of law said:

It made it easier to have a person responsible [the expert] for filling in the items. Someone who has the logic in mind, who knows what to write in which area, and then to discuss it with the people involved in the project. I'm not sure I'm able to fill in the right items.

Professor of law 1

Although the methodology was seen as more easily usable if an expert was present, some team members thought that it could nevertheless serve as a guideline for teams with no prior experience in serious game development:

I was involved, but a lot of it was handled by [the expert]. I don't know if I would have been able to do this. But it would have been a good base, a checklist.

Professor of law 2

I especially have questions: Without the coaching we received, would we have been able to follow the method with the same efficiency? I think that it is a very interesting tool, but there is still the need to have someone who translates the issues behind the method.

Project Owner

Some team members said that, in future projects, while gaining experience, they would be interested in using the co.LAB framework more independently:

In future projects, I would be really interested in doing it individually. But I'd also be interested in having someone behind me telling me if I did it right or wrong. I don't know if I would be able to, but when I see the methodology, it looks simple; it structures a project. I'd be interested in trying to use it independently.

Developer 1

#### Team Dynamics During the Focus Group

While speaking about the use of the co.LAB framework for teamwork and collaboration, team members mostly agreed with each other. The agreement with what was already said was sometimes made explicit with the use of expressions like “I agree with…” and “I’m joining what was said.” Even though it was not explicit at other moments, content analysis still showed convergent opinions. Content similarity was stronger among participants who had the same functions in the team, while the project owner expressed several specific points of view. 

There was no explicit disagreement between team members during the discussion. Team members never confronted each other directly but different points of view were expressed, particularly regarding the personal use of the framework and how they felt about using it in autonomy for further projects. 

Participants raised some questions about the use of the framework in other team configurations and wondered whether its use would also be appropriate for larger development teams.

### Questionnaires

We sent 11 emails to members of development teams belonging to either of the 2 projects, all of whom completed the questionnaire (100%). They completed the questionnaire between June 29, 2021 and July 7, 2021. No questionnaire was excluded as our exclusion criterion was not met. Participants' characteristics are detailed in Table 2.

**Table 2:** Characteristics of the study participants.

Characteristics		PRITS^a^ project (n=6)	InterMIS^b^ project (n=5)
Age (years), median (Q1, Q3)		33 (33, 38)	38 (36, 38)
**Gender, n**			
	Female	4	1
	Male	2	4
	Other	0	0
**Role(s) in the project^c^, n**			
	Computer scientist	2	1
	Game designer	2	0
	Graphic designer	1	0
	Pedagogue	0	1
	Serious game expert	0	1
	Subject matter expert	1	4
	Teacher	3	0
**Level of experience in serious game development prior to project inception, n**			
	None	5	4
	Limited	1	0
	Intermediate	0	0
	High	0	1

^a^PRITS: Patients’ Rights and Innovative Teaching Strategies.

^b^InterMIS: Interprofessional Major Incident Simulator.

^c^Participants could have more than one role.

The following sections present the results of the questionnaire regarding the influence of the co.LAB Framework on the collaborative dimensions identified by Burkhardt et al [21] and then on the other specific items inferred from the FG analysis.

#### Fluidity of Collaboration

**Figure 2:**
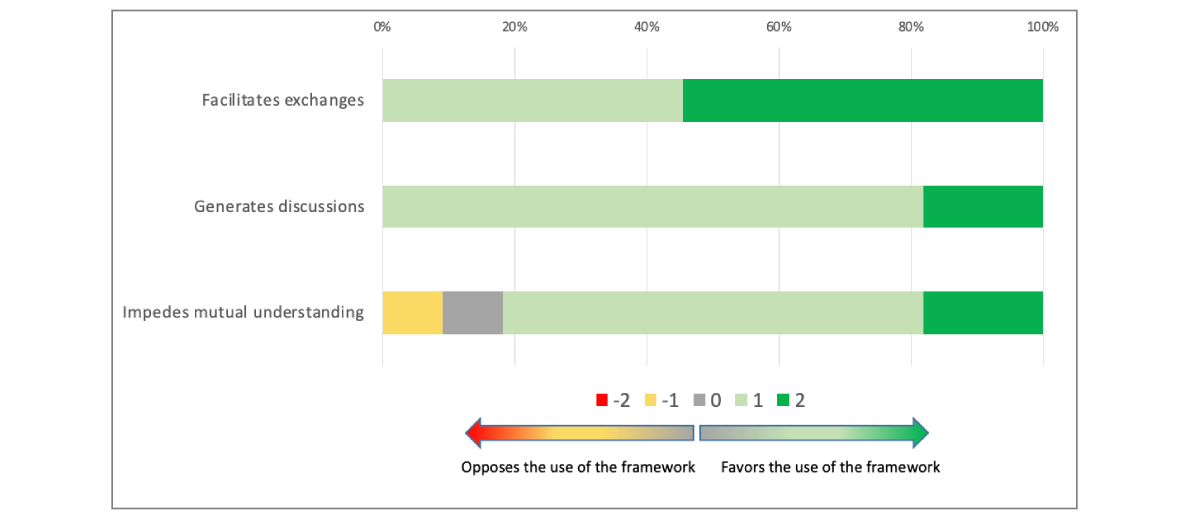
Assessment of fluidity of collaboration with the co.LAB framework.

According to the participants, the co.LAB framework facilitates exchange between specialists from different disciplines (median 2; Q1, Q3: 1, 2), helps generate discussion between these specialists (median 1; Q1, Q3: 1, 1), and does not impede mutual understanding (median 1; Q1, Q3: 1, 1). A graphical representation of these results is displayed in Figure 2.

The overall assessment of this dimension was in favor of the co.LAB framework (median 4; Q1, Q3: 3, 5), with no significant difference between groups (*P*=.06).

#### Sustaining Mutual Understanding

**Figure 3:**
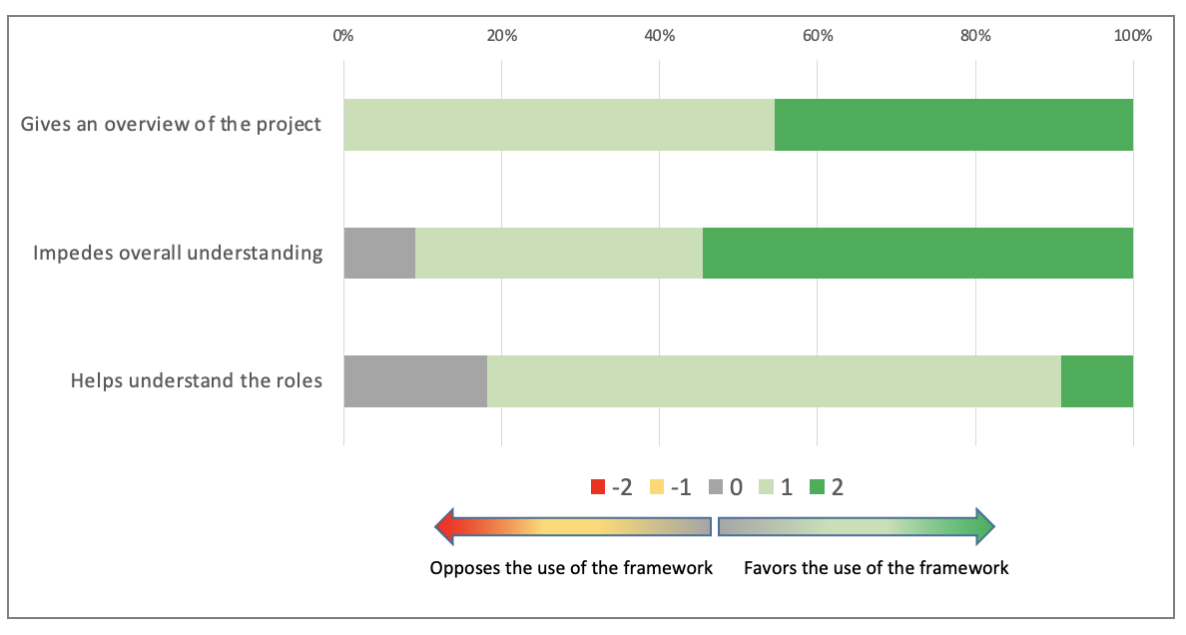
Sustaining mutual understanding with the co.LAB framework.

The co.LAB framework was considered to provide an overall view of the project (median 1; Q1, Q3: 1, 2). It also helped understand the roles of the different team members (median 1; Q1, Q3: 1, 1) and did not make overall understanding difficult (median 2; Q1, Q3: 1, 2). These results are shown in Figure 3.

Overall, this dimension was clearly supported by the use of the co.LAB framework (median 4; Q1, Q3: 3, 5). There was no statistically significant difference between the PRITS and InterMIS groups (*P*=.64).

#### Information Exchange for Problem Solving

**Figure 4:**
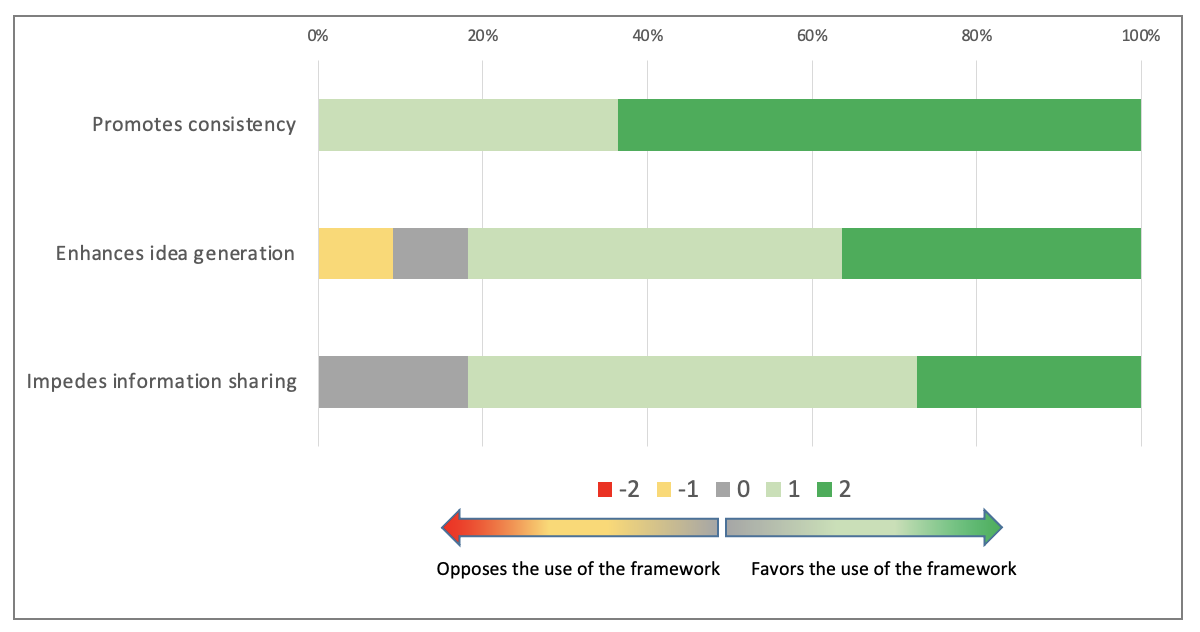
Information exchange for problem solving using the co.LAB framework.

The participants answered that using the co.LAB framework promoted consistency in the collaborative search for solutions (median 2; Q1, Q3: 1, 2) and enhanced idea generation (median 1; Q1, Q3: 1, 2). It was not considered to make information sharing more difficult (median 1; Q1, Q3: 1, 2; Figure 4).

Overall information exchange was therefore facilitated by the use of the co.LAB framework (median 4; Q1, Q3: 2, 5). PRITS and InterMIS participants provided similar answers (*P*=.52).

#### Argumentation and Reaching Consensus

**Figure 5:**
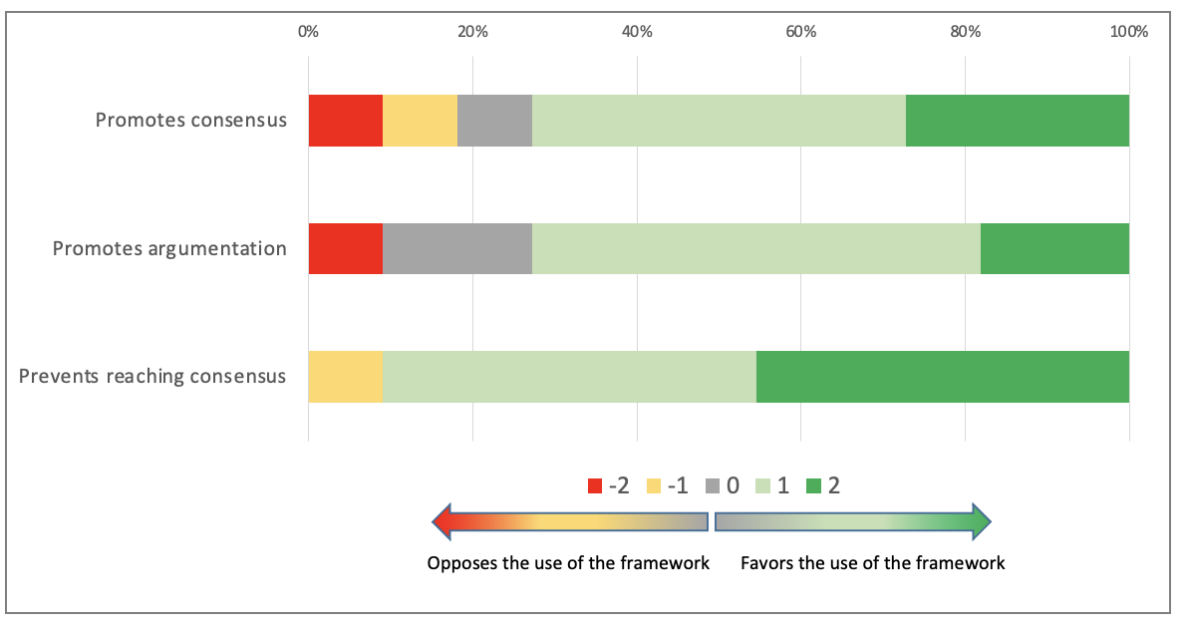
Argumentation and reaching consensus using the co.LAB framework.

Consensus building was promoted by the use of the co.LAB framework (median 1; Q1, Q3: 0, 2). This framework was also seen as promoting argumentation on alternative solutions (median 1; Q1, Q3: 0, 1) and was not considered as preventing reaching consensus (median 1; Q1, Q3: 1, 2; Figure 5).

Overall, this dimension was also supported by the use of the co.LAB framework (median 4; Q1, Q3: 2, 4) with no difference between groups (*P*=.70).

#### Task and Time Management

**Figure 6:**
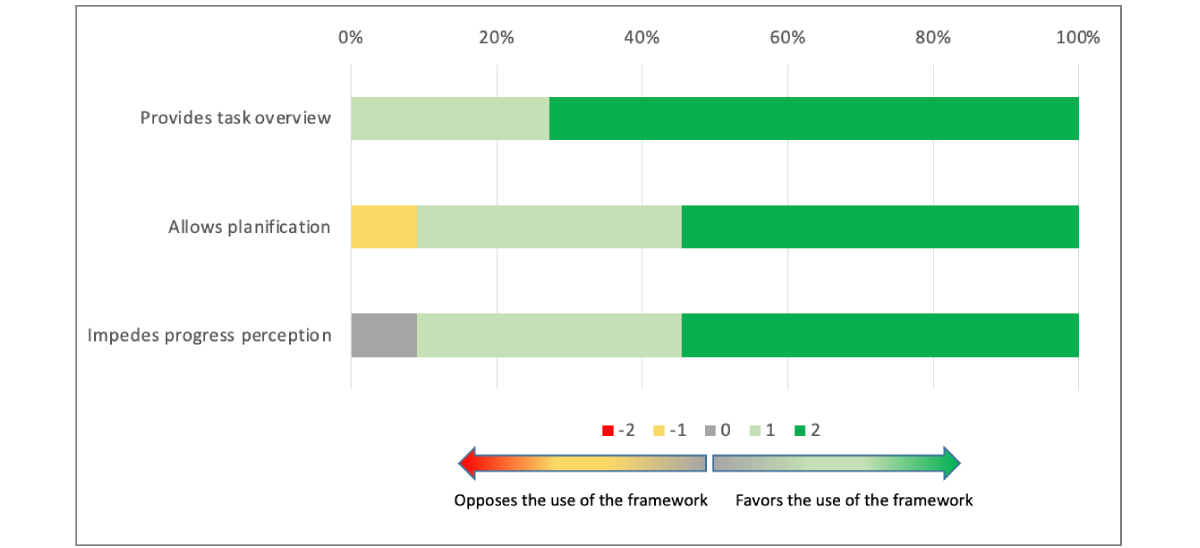
Task and time management using the co.LAB framework.

Participants generally agreed that the co.LAB framework was effective at providing an overview of the work to be achieved (median 2; Q1, Q3: 1, 2) and allowed planning of tasks (median 2; Q1, Q3: 1, 2). They did not think that its use made it difficult to understand the progress of the project (median 2; Q1, Q3: 1, 2; Figure 6).

Overall, this dimension received high ratings (median 5; Q1, Q3: 3, 6), with no difference between groups (*P*=.40).

#### Cooperative Orientation

**Figure 7:**
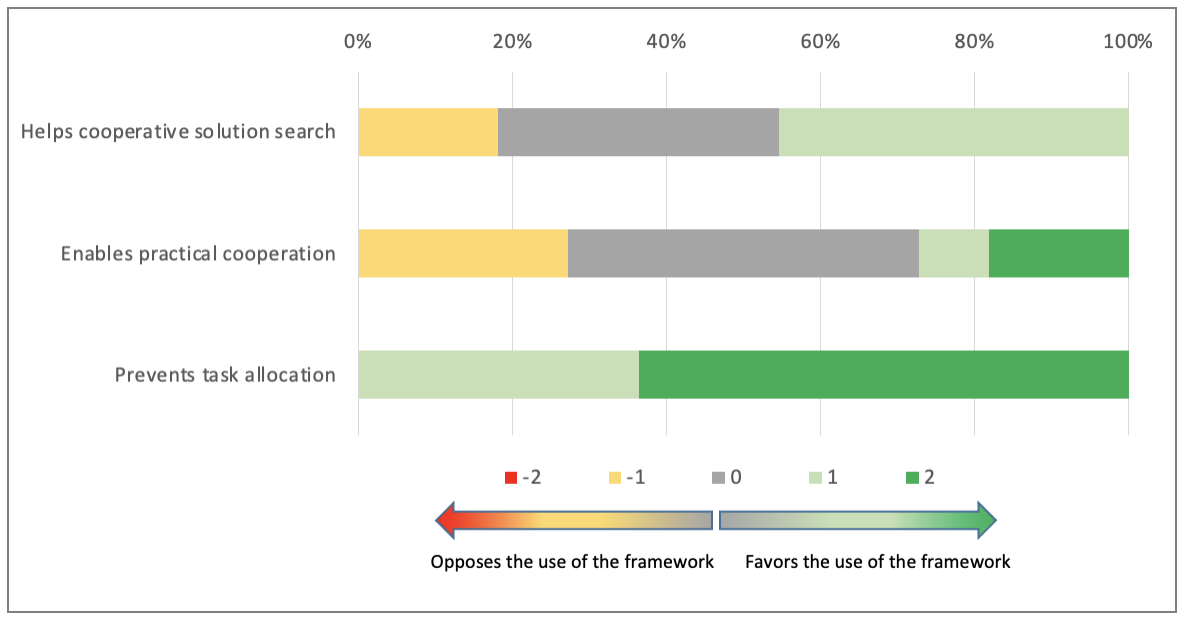
Cooperative orientation using the co.LAB framework.

Participants were less convinced by the usefulness of the co.LAB framework regarding the promotion of equal contributions in the search for a solution (median 0; Q1, Q3: 0, 1) or in achieving solutions (median 0; Q1, Q3: –1, 1). They were, however, convinced that using this framework did not interfere with task distribution (median 2; Q1, Q3: 1, 2; Figure 7).

The overall analysis of this dimension nevertheless favored the use of the co.LAB framework (median 2; Q1, Q3: 1, 4), with no statistically significant difference between groups (*P*=.09).

#### Individual Task Orientation

**Figure 8:**
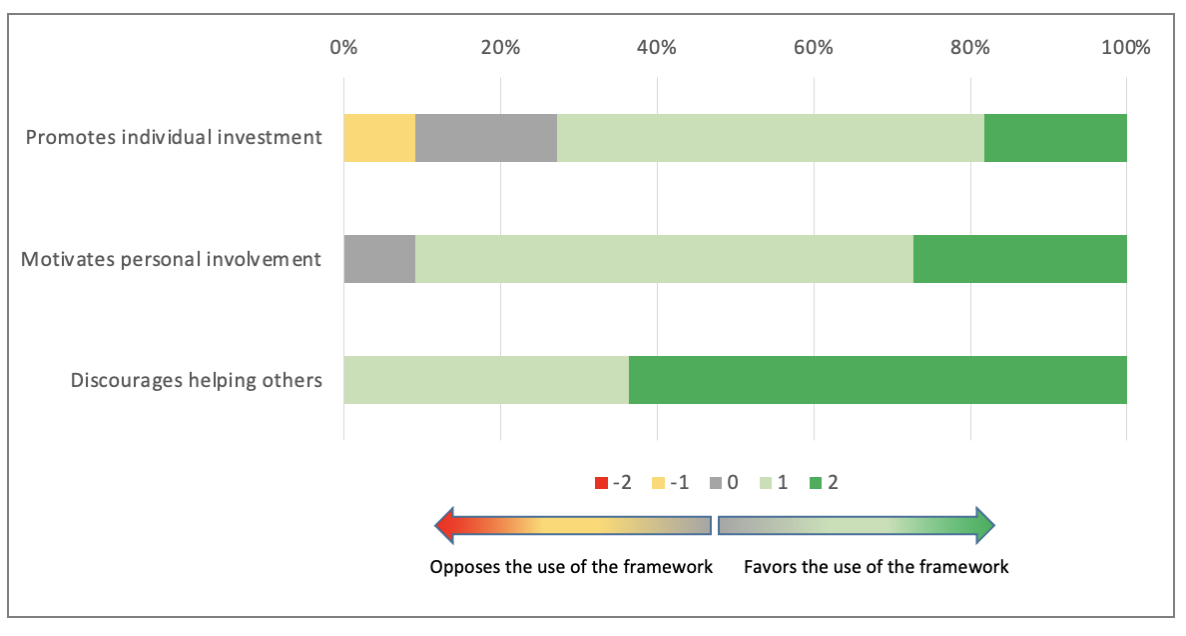
Individual task orientation using the co.LAB framework.

The co.LAB framework was considered as efficient in promoting individual investment throughout the project (median 1; Q1, Q3: 0, 1) and in motivating personal involvement (median 1; Q1, Q3: 1, 2). Participants who used the framework were not less prone to help others (median 2; Q1, Q3: 1, 2; Figure 8).

The overall analysis of this last dimension also supported the use of the co.LAB framework (median 3; Q1, Q3: 2, 5). There was no difference between groups (*P*=.08).

#### Overall Assessment

**Figure 9:**
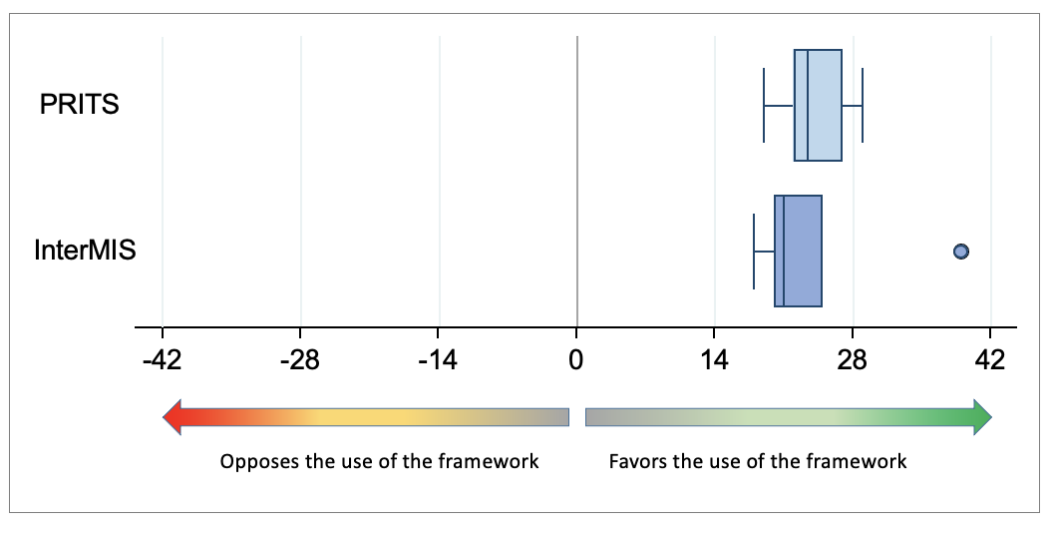
Overall assessment of the 7 dimensions of collaboration. InterMIS: Interprofessional Major Incident Simulator; PRITS: Patients’ Rights and Innovative Teaching Strategies.

Figure 9 shows the results obtained after pooling the 21 questions used to assess the 7 collaborative dimensions by Burkhardt et al [21].

No statistically significant difference was detected (*P*=.58). Figure 9 shows that there was a clear outlier in the InterMIS group, who rated the co.LAB framework much higher than all other participants. This outlier was a subject matter expert who had no prior experience in serious game development.

#### Collaboration Items Specific to the co.LAB Framework

All participants agreed that the co.LAB framework represents a guide for the design and development of serious games (median 2; Q1, Q3: 1, 2), with no difference between groups (*P*=.99). One participant commented that any comparison would be difficult without knowing any other framework or method. Another highlighted the fact that the framework “does not give the solution but structures the different steps necessary to the development [of the serious game].” The 2 participants who had prior experience in serious game design and development said that they had not used a specific framework or method during their previous venture. Both rated their experience with the co.LAB framework as either positive or very positive in comparison with their prior development.

Most participants thought that, for a team without prior experience in serious game design, a serious game expert would be mandatory to use the framework (median 2; Q1, Q3: 1, 2). A participant commented that “it [was] mandatory” while another was of the opinion that “it might not be mandatory, but it certainly has an added value.” In line with this latter comment, participants were not convinced that the co.LAB framework could serve as a standalone guide for a team without prior experience and without an expert (median 0; Q1, Q3: –1, 1). However, all participants were convinced that having a person responsible for summarizing the information regarding the project and its progress was necessary regardless of the experience of the development team (median 2; Q1, Q3: 1, 2).

The effect of the co.LAB framework on the quality of the collaboration during the design and development of the serious game was highly rated (median 4; Q1, Q3: 4, 4), with no difference between groups (*P*=.99). Participants rated even higher the impact of personal characteristics (personalities, past experiences) on the quality of the collaboration during the design and development phase (median 5; Q1, Q3: 4, 5). Both groups gave similar ratings to this item (*P*=.99).

Finally, all participants were convinced that the co-LAB framework should be implemented in a collaborative web platform (median 4; Q1, Q3: 4, 5), with no significant difference between groups (*P*=.24).

## Discussion

### Principal Findings

#### Essential Findings

The co.LAB framework was conceived to facilitate the design and development of serious games through a collaborative, multidisciplinary, adaptive, systemic approach [9]. The main objective of the present study was to gain insight regarding the contribution of the co.LAB framework to the collaboration within multidisciplinary teams during serious game design and development. Overall results (FG and questionnaires) show that the co.LAB framework had a positive impact on collaboration within multidisciplinary teams during serious game design and development.

#### Impact on Collaboration

The qualitative analysis of the FG session shows that the co.LAB framework was perceived as having a positive influence on several dimensions of collaboration. Participants spontaneously noted the positive impact of the co.LAB framework on the fluidity of communication, mutual understanding, information sharing, argumentation, motivation, management of project activities, and monitoring. This positive influence was similarly noted by participants from different professional disciplines. One dimension was not addressed: the fair balance of verbal contributions and activities carried out by the actors. While participants shared the impression that the co.LAB framework enhanced multidisciplinary collaboration, they also felt that it helped them manage their own specific tasks. These were related either to the discipline or to their role (eg, the game developer using information for the design of interfaces or the project owner seeking information about the project progresses for the funders). Thus, we can assume that the co.LAB framework could simultaneously support and build a shared understanding, while providing individual team members with specific information necessary to carry out their activities. These findings reveal that the use of this framework was positively perceived by team members. Moreover, it allows them to overcome some difficulties such as mutual understanding and working with individuals from different fields, which have been described as simultaneously a necessary and challenging process [4].

The quantitative analysis yielded results consistent with those obtained through qualitative analysis. The use of the co.LAB framework was perceived to have a positive impact on all dimensions of the collaboration. The dimension that was the least impacted by the use of the framework was facilitation of equal contributions, both in the search for solutions and in the development of those solutions. This is consistent with the FG results, as this dimension was the only one not mentioned by the participants during the session. We hypothesize that the rather low impact of the co.LAB framework on the equality of contribution could be explained by the fact that the framework seeks to give an overview of the solution to be developed but does not give any guidelines on the distribution of contributions. Moreover, we believe that the design and development of a serious game by a multidisciplinary team does not necessarily imply a proportional distribution of contributions. For example, a computer scientist may have to work several months on design and development, but a professional expert may spend only 1 day on the validation of knowledge foundations.

Collaboration has previously been described as a challenging but necessary process for serious game conception [4]. Overall qualitative and quantitative results of this study support the hypothesis that the use of a visual design framework, such as the co.LAB framework, provides an overview that enables both mutual understanding and the collaborative development of an integrated and coherent solution. In addition, a positive impact on time and task management was also reported. Thus, overall results suggest that the co.LAB framework helps overcome some of the inherent challenges linked with collaboration during serious game design and development.

#### Need for a Serious Game Expert

This study also revealed some additional points of interest regarding contingency factors influencing the use of the co.LAB framework. During the FG, an unexpected element was mentioned by participants: All of them agreed on the need to have a serious game development expert who understands the methodology and can guide the team. The results of the web-based questionnaire, which contained 3 questions designed to specifically assess this element, are consistent with this finding. There was no clear agreement on the possible use of the methodology without resorting to such an expert.

We hypothesize that this can be explained in the following way: The co.LAB framework defines “building blocks” (eg, learning objectives, pedagogical scenarios, game mechanics) that need to be designed and developed. Disciplinary skills are necessary for each of those domains (for example, a user experience [UX]/user interface [UI] specialist is needed to develop user interfaces). In the same way, the co.LAB framework provides guidelines for overall serious game design as these elements are not independent but interconnected [9]. Therefore, disciplinary skills in serious game design are needed to understand the interrelation between design elements and encourage coherence between them. This can also explain why the majority of team members, experts in their field but novices in serious game design, felt comfortable using the co.LAB framework in their field of expertise but not in all areas of serious game design. However, we believe that the co.LAB framework could allow novices to progressively develop expertise in serious game design and a better understanding of the other dimensions at stake. From a time perspective, some members expressed interest in developing serious game design expertise.

Therefore, we argue that a serious game design team should include disciplinary skills needed to cover each element of the serious game design and address the need for competencies related to the overall design and development process.

### Limitations

The first limitation of our study is related to its limited sample size, despite a very high participation rate. This could hardly be helped as, to date, only 2 projects have been developed with the methodology supported by the co.LAB framework. In addition, at the time of this study, both projects were going well in terms of both deadlines and quality of the results obtained. This may have had an impact on the perception of the contribution of the co.LAB framework but could also be seen as an effect of the use of the framework.

Furthermore, there are methodological limitations related to the use of FGs in qualitative research. Some of the recognized limitations for this type of method are the fact that some people speak more while others remain in the background (although this should have been counterbalanced by the moderation), and alignment of ideas, status, and roles can influence the opinions of the participants. To mitigate these limitations, some decisions were made. As one of the team members was the main author of the co.LAB framework, he did not participate as a participant in the FG but as an observer. As a second team member contributed to this study (ie, participated in the definition of the assessment of the contribution of the co.LAB framework to collaboration), she did not act as a participant but as a facilitator. Since she had a good understanding of the subject at hand, she was able to conduct the FG with valuable prior knowledge. Her experience as a researcher in qualitative research was another advantage in facilitating the FG.

Another limitation is related to the context of use of the co.LAB framework. In both projects, an expert of the co.LAB framework was part of the serious game development team. Therefore, this study does not give information regarding the possible use of the co.LAB framework without a methodological expert but rather shows that it can be useful for novices provided they receive appropriate guidance. It is not possible to state whether the importance of the presence of the expert was dependent on the framework itself or on team configuration, as in both projects, the same expert was part of the team. Further investigation is needed to measure the impact of this factor on team collaboration.

Finally, even though the questionnaire administered to participants was developed on the basis of the questionnaire by Burkhardt et al [21], it has not been validated. Given the small sample size, we refrained from performing a reliability analysis, and future studies including larger samples should be used for this purpose.

### Future Work

The co.LAB framework is currently being implemented in a web platform in which it is planned to embed questionnaires to evaluate its contribution to collaboration. This will allow for larger-scale validation.

Beyond assessing the impact of the framework on collaboration, this study revealed the perceived importance to the team of having a serious game expert. The analysis of the expert's contribution versus the framework's contribution, as well as the influence of the expert's personality, are topics for future research.

### Conclusion

Qualitative and quantitative results obtained through this study support the use of the co.LAB framework to facilitate collaboration within multidisciplinary serious game design and development teams.

In both projects included in this study, the co.LAB framework enhanced several dimensions of collaboration within multidisciplinary teams. However, expert guidance seems necessary to maximize development efficiency. Whether such guidance can be provided by means of a collaborative web platform remains to be determined.

## Appendix

Multimedia Appendix 1 COREQ: Consolidated Criteria for Reporting Qualitative Research.

Multimedia Appendix 2 Focus group interview guide translated.

Multimedia Appendix 3 Cherries checklist.

Multimedia Appendix 4 Web-based questionnaire translated.

## Abbreviations

CHERRIES: Checklist for Reporting Results of Internet E-Surveys
co.LAB: A Digital Lab for the co-Design, co-Development and co-Evaluation of Digital Learning Games
COREQ: Consolidated Criteria for Reporting Qualitative Research
FG: focus group
HESAV: School of Health Sciences
HES-SO: University of Applied Sciences of Western Switzerland
InterMIS: Interprofessional Major Incident Simulator
MEI: Media Engineering Institute
PRITS: Patients’ Rights and Innovative Teaching Strategies
UI: user interface
UX: user experience
