# Supplementary material for: Contribution of the co.LAB Framework to the Collaborative Design of Serious Games: Mixed Methods Validation Study
Source: JMIR Serious Games. 2021 Nov 24;9(4):e33144. doi: 10.2196/33144 (PMC8663511; doi:10.2196/33144)
Supplement: Multimedia Appendix 2 [file games_v9i4e33144_app2.pdf]

## Focus Group Interview Guide

| Field | | Original questions (in French) | English translation |
|---|---|---|---|
| Participants' background | 1.1 | Prenez une minute chacun pour vous présenter et présenter votre parcours en lien avec les serious games (expérience préalable dans les serious game, formation en lien avec les serious game, rôle principal dans la conception du jeu PRITS). | Take one minute each to present yourself and your background related to serious games (previous experience in serious games, training related to serious games, main role in the design of the PRITS game). |
| First use of the co.LAB framework | 1.2 | Quand avez-vous été amenés à utiliser la méthodologie co.LAB pour la 1ère fois ? | When did you first come to use the co.LAB methodology? |
| Use of the co.LAB framework | 1.3 | Comment l'avez-vous utilisée dans votre travail ? | How did you use it in your work? |
| Evaluation of the co.LAB framework | 2. | Quels éléments de la méthodologie co.LAB avez-vous particulièrement appréciés ? | What elements of the co.LAB methodology did you particularly appreciate? |
| Impact on collaboration | 2.1 | Ces éléments ont-ils affecté le travail collaboratif ?<br><br>Par exemple, la méthodologie a-t-elle permis de soutenir :<br>● fluidité de la collaboration<br>● compréhension mutuelle<br>● échange d'information pour la résolution de problèmes<br>● argumentation et atteinte d'un consensus<br>● gestion des tâches et du temps<br>● la coopération entre les membres<br>● la motivation et l'engagement individuel dans les tâches | Have these elements affected collaborative work?<br><br>For example, did the framework support:<br><br>● fluidity of the collaboration<br>● mutual understanding<br>● information exchange for problem solving<br>● argumentation and consensus building<br>● task and time management<br>● cooperation between members<br>● motivation and individual commitment to tasks |
| Impact on work | 2.2 | Y'a-t-il eu d'autres effets sur le travail ? | Were there any other effects on the work? |
| Evaluation of the co.LAB methodology | 2.3 | Quels éléments de la méthodologie avez-vous moins appréciés ? | What elements of the methodology did you like less? |

Supplementary Table 1 – Original focus group questions (in French) and English translation

| | | | |
|---|---|---|---|
| Improvements | 3.1 | AlbaSim[1] désire poursuivre le développement de la méthodologie co.LAB pour la conception de serious game, Quels conseils avez-vous pour eux ?<br>● Qu'est-ce qu'il faut garder ?<br>● Qu'est-ce qui pourrait être amélioré ? | AlbaSim wants to pursue the development of the co.LAB methodology for serious game design. What advice do you have for them?<br>● What should be kept?<br>● What could be improved? |
| Web plateform | 3.2 | AlbaSim désire poursuivre le développement de la méthodologie co.LAB notamment en créant une plateforme web, qu'en pensez-vous ? (Intérêt, fonctionnalité, difficultés) | AlbaSim wishes to continue the development of the co.LAB methodology, by creating a web platform, what do you think? (interest, functionality, difficulties) |
| Summary | 4 | Résumé par la modératrice | Summary by the moderator |
| Summary's adequacy | 4.1 | Est-ce que c'est un résumé adéquat ? | Is this an adequate summary? |
| Closing | 4.2 | Y'a-t-il quelque chose que nous aurions dû discuter mais que nous n'avons pas fait ? | Is there anything we should have discussed but did not? |

[1] AlbaSim is a research group from the Media Engineering Institute (MEI)
