# Supplementary material for: Contribution of the co.LAB Framework to the Collaborative Design of Serious Games: Mixed Methods Validation Study
Source: JMIR Serious Games. 2021 Nov 24;9(4):e33144. doi: 10.2196/33144 (PMC8663511; doi:10.2196/33144)
Supplement: Multimedia Appendix 3 [file games_v9i4e33144_app3.pdf]

Item Category	*Checklist Item*	*Explanation*	*Comment*
**Design**	Describe survey design	Describe target population, sample frame. Is the sample a convenience sample? (In "open" surveys this is most likely.)	"The questionnaire was administered to a convenience sample of eleven participants: six from the PRITS project and five from the InterMIS project."
**IRB (Institutional Review Board) approval and informed consent process**	IRB approval	Mention whether the study has been approved by an IRB.	This was not a medical study. Therefore, there was no need for an IRB approval. In addition, the population studied is not considered as "vunerable" according to Swiss Federal Law.
	Informed consent	Describe the informed consent process. Where were the participants told the length of time of the survey, which data were stored and where and for how long, who the investigator was, and the purpose of the study?	"Participants provided their consent to the recording and use of the data for research purposes."
	Data protection	If any personal information was collected or stored, describe what mechanisms were used to protect unauthorized access.	"The web platform was protected by two different software firewalls: RSFirewall (RSJoomla, Constanta, Romania) and Admin Tools (Akeeba Ltd., Nicosia, Cyprus)." and "All data was securely recorded and stored in an encrypted mySQL-compatible database (MariaDB 5.5.5, MariaDB Foundation, Delaware, USA) hosted on a Swiss server."
**Development and pre-testing**	Development and testing	State how the survey was developed, including whether the usability and technical functionality of the electronic questionnaire had been tested before fielding the questionnaire.	"Based on FG results, a three-tiered web-based questionnaire was developed following Eysenbach's Checklist for Reporting Results of Internet E-Surveys (CHERRIES)" and "The platform, the registration form, the questionnaire and the data extraction mechanism were thoroughly tested by all coauthors before inviting participants to complete the questionnaire."
**Recruitment process and description of the sample having access to the questionnaire**	Open survey versus closed survey	An "open survey" is a survey open for each visitor of a site, while a closed survey is only open to a sample which the investigator knows (password-protected survey).	"[The questionnaire was] administered to a convenience sample of eleven participants: six from the PRITS project and five from the InterMIS project."
	Contact mode	Indicate whether or not the initial contact with the potential participants was made on the Internet. (Investigators may also send out questionnaires by mail and allow for Web-based data entry.)	See above
	Advertising the survey	How/where was the survey announced or advertised? Some examples are offline media (newspapers), or online (mailing lists – If yes, which ones?) or banner ads (Where were these banner ads posted and what did they look like?). It is important to know the wording of the announcement as it will heavily influence who chooses to participate. Ideally the survey announcement should be published as an appendix.	"Only then were participants invited, by email, to complete the questionnaire."
**Survey administration**	Web/E-mail	State the type of e-survey (eg, one posted on a Web site, or one sent out through e-mail). If it is an e-mail survey, were the responses entered manually into a database, or was there an automatic method for capturing responses?	"[The survey] was created using the Community Surveys 5.6 component (Corejoomla, Hyberabad, India)" and "All data was automatically recorded [...]"

	Context	Describe the Web site (for mailing list/newsgroup) in which the survey was posted. What is the Web site about, who is visiting it, what are visitors normally looking for? Discuss to what degree the content of the Web site could pre-select the sample or influence the results. For example, a survey about vaccination on a anti-immunization Web site will have different results from a Web survey conducted on a government Web site	"The questionnaire was hosted on a specific web platform"
	Mandatory/voluntary	Was it a mandatory survey to be filled in by every visitor who wanted to enter the Web site, or was it a voluntary survey?	This is implicit: the website was only visited by those who were invited to complete the survey.
	Incentives	Were any incentives offered (eg, monetary, prizes, or non-monetary incentives such as an offer to provide the survey results)?	"No incentive was provided to encourage participants to complete the survey."
	Time/Date	In what timeframe were the data collected?	"They completed the questionnaire between 06.29.2021 and 07.07.2021"
	Randomization of items or questionnaires	To prevent biases items can be randomized or alternated.	Not relevant for this kind of study
	Adaptive questioning	Use adaptive questioning (certain items, or only conditionally displayed based on responses to other items) to reduce number and complexity of the questions.	This is described in Table 1: "Branching logic was used to display this question only when relevant according to prior responses"
	Number of Items	What was the number of questionnaire items per page? The number of items is an important factor for the completion rate.	This is thoroughly detailed in Table 1
	Number of screens (pages)	Over how many pages was the questionnaire distributed? The number of items is an important factor for the completion rate.	This is thoroughly detailed in Table 1
	Completeness check	It is technically possible to do consistency or completeness checks before the questionnaire is submitted. Was this done, and if “yes”, how (usually JAVAScript)? An alternative is to check for completeness after the questionnaire has been submitted (and highlight mandatory items). If this has been done, it should be reported. All items should provide a non-response option such as “not applicable” or “rather not say”, and selection of one response option should be enforced.	"Multiple answer and multiple choice questions were mandatory and had to be completed before participants were allowed to proceed to the next page."
	Review step	State whether respondents were able to review and change their answers (eg, through a Back button or a Review step which displays a summary of the responses and asks the respondents if they are correct).	"Answers could be modified until the questionnaire was marked as completed (after clicking the “Finish” button). Questionnaires could be resumed if the participant was disconnected or chose to log out temporarily."
**Response rates**	Unique site visitor	If you provide view rates or participation rates, you need to define how you determined a unique visitor. There are different techniques available, based on IP addresses or cookies or both.	"To avoid potential double-entries, participants were required to fill in a short registration form before they could access the survey."
	View rate (Ratio of unique survey visitors/unique site visitors)	Requires counting unique visitors to the first page of the survey, divided by the number of unique site visitors (not page views!). It is not unusual to have view rates of less than 0.1 % if the survey is voluntary.	Not relevant for this study

	Participation rate (Ratio of unique visitors who agreed to participate/unique first survey page visitors)	Count the unique number of people who filled in the first survey page (or agreed to participate, for example by checking a checkbox), divided by visitors who visit the first page of the survey (or the informed consents page, if present). This can also be called "recruitment" rate.	Not relevant for this study
	Completion rate (Ratio of users who finished the survey/users who agreed to participate)	The number of people submitting the last questionnaire page, divided by the number of people who agreed to participate (or submitted the first survey page). This is only relevant if there is a separate "informed consent" page or if the survey goes over several pages. This is a measure for attrition. Note that "completion" can involve leaving questionnaire items blank. This is not a measure for how completely questionnaires were filled in. (If you need a measure for this, use the word "completeness rate".)	"Eleven emails were sent to potential participants, all of whom completed the questionnaire (100%)."
**Preventing multiple entries from the same individual**	Cookies used	Indicate whether cookies were used to assign a unique user identifier to each client computer. If so, mention the page on which the cookie was set and read, and how long the cookie was valid. Were duplicate entries avoided by preventing users access to the survey twice; or were duplicate database entries having the same user ID eliminated before analysis? In the latter case, which entries were kept for analysis (eg, the first entry or the most recent)?	Not relevant as a registration form was used.
	IP check	Indicate whether the IP address of the client computer was used to identify potential duplicate entries from the same user. If so, mention the period of time for which no two entries from the same IP address were allowed (eg, 24 hours). Were duplicate entries avoided by preventing users with the same IP address access to the survey twice; or were duplicate database entries having the same IP address within a given period of time eliminated before analysis? If the latter, which entries were kept for analysis (eg, the first entry or the most recent)?	Not relevant as a registration form was used.
	Log file analysis	Indicate whether other techniques to analyze the log file for identification of multiple entries were used. If so, please describe.	Not relevant as a registration form was used.
	Registration	In "closed" (non-open) surveys, users need to login first and it is easier to prevent duplicate entries from the same user. Describe how this was done. For example, was the survey never displayed a second time once the user had filled it in, or was the username stored together with the survey results and later eliminated? If the latter, which entries were kept for analysis (eg, the first entry or the most recent)?	"To avoid potential double-entries, participants were required to fill in a short registration form before they could access the survey."

**Analysis**	Handling of incomplete questionnaires	Were only completed questionnaires analyzed? Were questionnaires which terminated early (where, for example, users did not go through all questionnaire pages) also analyzed?	Not applicable as all questionnaires were duly completed
	Questionnaires submitted with an atypical timestamp	Some investigators may measure the time people needed to fill in a questionnaire and exclude questionnaires that were submitted too soon. Specify the timeframe that was used as a cut-off point, and describe how this point was determined.	"Questionnaires containing inconsistent answers were excluded. Inconsistent answers were detected when either the maximum (5) or minimum value (1) was given to all three questions assessing a specific dimension of collaboration as each dimension contained inversely phrased questions."
	Statistical correction	Indicate whether any methods such as weighting of items or propensity scores have been used to adjust for the non-representative sample; if so, please describe the methods.	"All questions were evenly weighted."
