# Supplementary material for: Contribution of the co.LAB Framework to the Collaborative Design of Serious Games: Mixed Methods Validation Study
Source: JMIR Serious Games. 2021 Nov 24;9(4):e33144. doi: 10.2196/33144 (PMC8663511; doi:10.2196/33144)
Supplement: Multimedia Appendix 4 [file games_v9i4e33144_app4.pdf]

Web-based questionnaire

Page	Field	Original questions (in French)	Question (English translation)
1	Introduction	N/A	N/A
2	Demographics and role in the project	A quel genre vous identifiez-vous?	What gender do you identify with? [1]
		Quel est votre âge?	How old are you? [2]
		Dans la conception du serious game, ma(mes) fonction(s) principale(s) est	Main roles during the current serious game design project[3]
		Au début de ce projet, quelle était votre expérience dans le design et développement de serious games ?	At the start of this project, what was your experience in the design and development of serious games? [1]
3	Fluidity of collaboration [4]	La méthodologie co.LAB facilite les échanges entre les spécialistes des différentes disciplines	The co.LAB framework facilitates exchanges between specialists from different disciplines [1,5]
		La méthodologie co.LAB génère les discussions entre spécialistes des différentes disciplines	The co.LAB framework generates discussions between specialists from different disciplines [1,5]
		La méthodologie co.LAB rend la compréhension mutuelle difficile	The co.LAB framework impeded mutual understanding [1,5,6]
	Sustaining mutual understanding [4]	La méthodologie co.LAB donne une vue d'ensemble de la solution à développer	The co.LAB framework gives an overview of the solution to be developed [1,5]
		La méthodologie co.LAB rend difficile la compréhension globale	The co.LAB framework makes overall understanding difficult [1,5,6]

		La méthodologie co.LAB permet de comprendre les rôles des différents membres de l'équipe	The co.LAB framework helps to understand the roles of the different team members [1,5]
4	Information exchange for problem solving [4]	La méthodologie co.LAB favorise la cohérence dans la recherche collaborative de solutions	The co.LAB framework promotes consistency in the collaborative search for solutions [1,5]
		La méthodologie co.LAB favorise la génération d'idées	The co.LAB framework enhances the generation of ideas [1,5]
		La méthodologie co.LAB rend difficile le partage d'information	The co.LAB framework makes it difficult to share information [1,5,6]
	Argumentation and reaching consensus [4]	La méthodologie co.LAB favorise la recherche de consensus	The co.LAB framework promotes consensus building [1,5]
		La méthodologie co.LAB favorise l'argumentation sur les variantes de solutions	The co.LAB framework promotes argumentation on alternative solutions [1,5]
		La méthodologie co.LAB empêche l'atteinte d'un consensus	The co.LAB framework prevents reaching consensus [1,5,6]
5	Task and time management [4]	La méthodologie co.LAB fournit une vue d'ensemble du travail à effectuer	The co.LAB framework provides an overview of the work to be achieved [1,5]
		La méthodologie co.LAB permet de planifier le travail à réaliser	The co.LAB framework allows task planification [1,5]
		La méthodologie co.LAB rend difficile la compréhension de l'avancement du projet	The co.LAB framework makes it difficult to understand the progress of the project [1,5,6]
	Cooperative orientation [4]	La méthodologie co.LAB favorise l'égalité des contributions dans la recherche de solution	The co.LAB framework promotes equal contributions in the search for a solution [1,5]
		La méthodologie co.LAB favorise l'égalité des contributions dans la réalisation des solutions	The co.LAB framework promotes equal contributions in achieving solutions [1,5]

		La méthodologie co.LAB nuit à la répartition du travail à réaliser	The co.LAB framework interferes with the distribution of the work to be done [1,5,6]
6	Individual task orientation [4]	La méthodologie co.LAB favorise un investissement individuel tout au long du projet	The co.LAB framework promotes individual investment throughout the project [1,5]
		La méthodologie co.LAB motive à s'impliquer personnellement dans le projet	The co.LAB framework motivates to become personally involved in the project [1,5]
		La méthodologie co.LAB ne donne pas envie d'aider les autres	The co.LAB framework does not make you want to help others [1,5,6]
7		Lors de votre dernière conception de serious game, avez-vous utilisé une méthode de design et développement ?	During your last serious game design, did you use a design and development method? [1]
		Quelle était la méthode utilisée ?	Which method was it? [7,8]
		En comparaison avec votre précédente expérience de design et développement de serious game, quel est votre perception de l'apport de co.LAB à la collaboration au sein de l'équipe ?	In comparison with your previous experience in serious game design and development, what is your perception of the contribution of co.LAB to collaboration within the team? [1,9]
8	Further elements of collaboration	La méthodologie co.LAB constitue un guide pour le design et le développement d'un serious game	The co.LAB framework represents a guide for the design and development of a serious game [1,5,10]
9		Pour une équipe sans expérience dans la conception de serious game, avoir une personne qui guide l'utilisation de la méthodologie co.LAB est nécessaire	For a team without prior experience in serious game design, a co.LAB expert is mandatory to use the framework [1,5,10]
10		Pour une équipe sans expérience et sans expert, la méthodologie co.LAB peut servir de guide sans avoir recours à un expert externe	For a team without prior experience and without an expert, the co.LAB framework can serve as a guide without having recourse to an external specialist. [1,5,10]

11		Quelle que soit l'expérience de l'équipe de design et développement, avoir une personne responsable d'effectuer la synthèse des informations et de l'avancement du projet est nécessaire	Regardless of the experience of the design and development team, having a person responsible for summarizing the information about project and its progress is mandatory [1,5,10]
12	Autonomy in using the co.LAB framework	Lors de la conception du serious game, à quel point estimez-vous être autonome dans l'utilisation de la méthodologie co.LAB ?	When designing the serious game, how autonomous did you feel in using the co.LAB framework? [1,10]
		Après avoir utilisé la méthodologie co.LAB dans un premier projet, à quel point estimez-vous être autonome dans l'utilisation de la méthodologie pour un nouveau projet ?	After using the co.LAB framework in a first project, how autonomous do you think you are in using this framework for another project? [1,10]
13	Final elements of collaboration	Quel poids donnez-vous à l'effet de la méthodologie co.LAB sur la qualité de la collaboration lors de la conception du serious game ?	What weight do you give to the effect of the co.LAB framework on the quality of the collaboration during the design of the serious game? [1,11]
		Quel poids donnez-vous à l'effet des caractéristiques personnelles des membres de l'équipe (personnalités, expériences, etc.) sur la qualité de la collaboration lors de la conception du serious game ?	What weight do you give to the effect of the personal characteristics of the team members (personalities, experiences, etc.) on the quality of the collaboration during the design and development of the serious game? [1,11]
14	Implementation in a web platform	Quel intérêt voyez-vous à l'implémentation de la méthodologie co.LAB dans une plateforme web collaborative pour la conception de serious games ?	What interest do you see in the implementation of the co.LAB framework in a collaborative web platform? [1,11]
15	Thank you page	N/A	N/A

N/A: not applicable;

[1] Multiple choice question (only one possible answer)

[2] Free text with Regex (regular expression) validation

[3] Multiple answer question (more than one possible answer)

[4] Dimension of collaboration according to Burkhardt et al. (REF)

[5] 5-point Likert scale ranging from “Stronly agree” to “Strongly disagree”

[6] Question negatively formulated to assess consistency

[7] Free text field

[8] Branching logic was used to display this question only when relevant according to prior responses

[9] 5-point Likert scale ranging from “Very positive impact” to “Very negative impact”

[10] A multi-line free text field was displayed below this question to allow participants to enter comments

[11] 5-point Likert scale ranging from “Very little / none” to “Very high”
